# “Discovering shine through feedback seeking”---feedback seeking among new graduate nurses: a qualitative study

**DOI:** 10.1186/s12912-023-01657-3

**Published:** 2024-01-02

**Authors:** Ziling Song, Yuanyuan Shen, Xin Yao, Siqi Wen, Jing Wang, Yanyan Chen, Peihua Zhang, Xiaoqiong Huang

**Affiliations:** 1 School of Ophthalmology and Optometry, Wenzhou Medical University, 270 West Xueyuan Road Wenzhou, Wenzhou, 325027 Zhejiang China; 2 School of Nursing, Wenzhou Medical University, Wenzhou, 325000 Zhejiang China; 3 National Clinical Research Center for Ocular Diseases, Eye Hospital, Wenzhou Medical University, 270 West Xueyuan Road, Wenzhou, 325027 Zhejiang China

**Keywords:** Nurses, Psychology, Feedback, Experience, Nursing research, Qualitative study

## Abstract

**Background:**

Feedback is critical to improving practitioners’ clinical practice and professional growth. Although they are still considered junior practitioners, their feedback-seeking experiences have yet to be investigated. This study aimed to understand the fundamental thoughts and experiences of new graduate nurses regarding feedback-seeking and to identify the main factors that influence their feedback-seeking behaviors.

**Methods:**

Conducting a descriptive phenomenological study, semi-structured in-depth interviews with newly graduated nurses from four hospitals in Zhejiang Province, China, face-to-face or via video call in the hospital conference room through purposive and snowball sampling. Interview data were evaluated using Colaizzi’s 7-step phenomenological data analysis. The COREQ checklist was followed.

**Results:**

A total of 15 new graduate nurses were interviewed as a sample, and 13 categories emerged from our data. They were categorized into four central elements: (1) perceptions and attitudes, (2) drivers, (3) dilemmas and needs, and (4) transformation and growth.

**Conclusions:**

This study found that new graduate nurses have various needs but face dilemmas in the feedback-seeking process. Nursing managers should be proficient at providing positive leadership, collaborating with clinical mentors to foster an atmosphere where new graduate nurses may obtain honest, transparent, and fair feedback, and exercising caution when providing negative feedback.

## Background

Global healthcare systems face enormous challenges due to care shortages caused by high turnover rates [1]. The mobility of nurses due to rising nursing workforce obligations fromglobal aging and increasingly complicated healthcare requirements is exacerbating the worldwide nursing shortage. Nurse turnover rates in several countries, such as Australia, the United States, and New Zealand, range from 12% to 60% [2]. The turnover rate for new graduate nurses(NGNs) in Korea is 42.7% [3], whereas in China, which has a severe shortage of nurses, 71.8% of NGNs tend to quit their jobs within the first year [4]. Moreover, psychological issues during COVID-19 led several nurses to resign or reevaluate their career choices [5]. The difficulties are significantly greater for NGNs in this circumstance [6]. According to a study [7], to ease the nursingworkforce shortage, guarantee a sufficient and qualified future workforce, and stabilize the nursing workforce, particular consideration should be given to NGNs and their well-being.

By actively seeking helpful information from a business, one engages in feedback-seeking behavior (FSB), which allows one to adjust to the organization’s demands and personal growth [8]. The connection between high occupational stress and poor health and job satisfaction affects workforce stability and quality of care among NGNs, who confront higher levels of occupational stress than nurses with more years of experience [9]. According to another study, receiving feedback is related to greater job satisfaction, more innovative performance, and fewer plans to leave among workers [10]. Therefore, soliciting feedback can lessen transfer shock and allow faster adjustment to the clinical setting for NGNs [11, 12]. The need for newly graduating nurses to seek a solid knowledge foundation, flexibility, and creativity through feedback to assure the quality and safety of medical treatment in a healthcare setting with ever-expanding knowledge and nurse exit-recruitment cycles has become urgent [13].

An essential management tactic is to encourage NGNs to seek feedback. According to Henry [14], professionals must participate in feedback-seeking exercises to be self-directed lifelong learners, and this proactive behavior should be encouraged during training. Lifelong learning is essential for individuals to expand and update their knowledge and abilities continually and for businesses to build and sustain a competitive advantage [15]. In this regard, seeking feedback is a powerful tool for personal growth and acquiring new knowledge and insulation. The ability of NGNs to gather further information and observations is especially essential to offering appropriate assurance of care quality and patient safety [16].

The study discovered that the clinical faculty’s supervisory role (supporting vs. instrumental leadership style) influenced surgical residents’ views of feedback’s advantages, costs, and subsequent FSBs [17]. However, more studies must be conducted on the factors influencing the FSB of NGNs. As a new force in the nursing team and a critical management target of the tendency to leave, NGNs urgently need to pay attention to the importance of their FSBs and the factors influencing them. In addition, comparing past studies by scholars, which primarily focused on the delivery of feedback itself and the study of the relationship between the motivation for input and the feedback environment [18, 19], there is no adequate understanding of the factors related to the facilitation and hindrance of FSB in a range of work scenarios for NGNs, which will not be conducive to the refinement of the feedback strategy and the full realization of feedback by NGNs in their continuing education.

Therefore, this study explored NGNs’ feedback-seeking experience through qualitative research methods. It aims to identify the main factors affecting their feedback-seeking and create a favorable environment for NGNs.

## Methods

### Design

Semi-structured interviews were used in conducting this descriptive phenomenological study. The study sought to improve our understanding through the perspective of NGNs who had witnessed the phenomenon [20]. A qualitative descriptive design was selected because this study sought to investigate NGNs’ feedback-seeking experiences.

### Respondents

From September 2022 to December 2022, NGNs from four hospitals in the Zhejiang Province were recruited for in-depth interviews using purposive and snowball sampling. The inclusion criteria were ① possession of a nursing license and ② 2 years of nursing work experience. The exclusion criteria were ① current work position being a non-clinical nursing post and ② current off-duty status, such as work and study leave. The authors first contacted two current nurses within 2 years of graduation at Hospital A using convenience and snowball sampling, explained the purpose and design of the study, and invited them to participate. Voluntary participation and personal anonymity were ensured. With permission to schedule a time for in-person interviews (Some were scheduled online during off hours due to COVID-19), all respondents received an informed consent form via email, which they were required to read in detail and sign. After the interview, participants were asked if they would be willing to assist the researcher in enquiring about other NGNs eligible for inclusion. Once the agreement was reached, contact details were shared with the researcher, and then a suitable time was chosen to interview potential NGNs at hospitals B, C, and D in the same manner described above. The saturation of the substance determined its sample size.

### Data collection

To acquire data, we conducted semi-structured interviews with NGNs. The participants for the guided interviews were chosen to collect information about the engagement of NGNs in feedback-seeking activities. The detailed interview outline is shown in Table 1. Respondents were questioned about their perspectives on feedback seeking, situations prompting the need to seek input, and their present issues and feedback requirements. The participants’ life experiences and opinions were then obtained using open-ended questions. Eventually, 15 NGNs were interviewed, and the general information of the interviewees is shown in Table 2. The interviews achieved saturation after 15 interviews since no novel participants surfaced. To rule out the likelihood of fresh knowledge exposure, the first author conducted two further interviews in late December 2022. In-person or online chat services were used to conduct interviews in Mandarin. The interview location is arranged in the meeting room of the department where the interviewee is located to ensure that the environment is private and quiet; the online interview meets the same conditions as above, adjusts the video equipment in advance, and is carried out when the network is stable, observes the interviewee’s facial expression, tone of voice, etc., and makes a good record. The interviews ranged in length from 45 to 60 min. During the communication process, the sequence and style of the interviews were flexibly altered based on the interviewees’ responses, and the valuable answers were suitably followed up on. The interviews were recorded and transcribed verbatim, and the entire process was recorded with the approval of the interviewees. The interview material was anonymized to protect the interviewees’ privacy.

**Table 1:** Interview outline for new graduate nurse feedback seeking experience

NO.	Questions
1	Do you actively seek feedback, and what do you think about seeking feedback?
2	How do you feel when seeking feedback?
3	What factors would motivate you to seek feedback?
4	What is your source of seeking feedback, and what are your suggestions regarding the current process?
5	What difficulties have you encountered in the process of seeking feedback? How did you resolve them?
6	What are the differences between student and post-work feedback seeking?

**Table 2:** General information of the interviewees (n = 15)

No.	Gender	Age	Education	Hospital	Interview Method
N1	Female	23	Undergraduate	A	Face to Face
N2	Female	22	Undergraduate	A	Face to Face
N3	Male	22	Undergraduate	A	Face to Face
N4	Male	23	Undergraduate	B	Video Call
N5	Female	22	Undergraduate	A	Face to Face
N6	Female	22	Undergraduate	A	Face to Face
N7	Female	23	Undergraduate	C	Video Call
N8	Female	23	Undergraduate	D	Video Call
N9	Female	22	Undergraduate	D	Video Call
N10	Female	23	Undergraduate	B	Video Call
N11	Female	22	Undergraduate	D	Video Call
N12	Female	24	Undergraduate	C	Video Call
N13	Female	23	Undergraduate	D	Face to Face
N14	Female	23	Undergraduate	D	Face to Face
N15	Male	24	Undergraduate	B	Face to Face

### Data analysis

To verify the accuracy and completeness of the data, the interviews were recorded and transcribed by two researchers independently within 24 hours after completion. The transcribed data were returned to the respondents to verify the stability and correctness of the data collection and research outcomes. The interview data were evaluated using Colaizzi’s 7-step phenomenological data analysis [21], which is detailed below. ①The two researchers practiced reading and arranging the original data by total transcription of all responses provided by respondents, including linguistic and nonverbal expressions (such as mannerisms, vocal pauses, and sighs). ② They identified and located meaningful statements by disassembling, analyzing, reassembling, and highlighting relevant data. ③Meaning construction is the process of creating or coding repeating ideas. ④Themes are clustered when similar or related codes are placed together to generate categories and subcategories. Manual coding was used in conjunction with the Nvivo 11 program. ⑤We provided a complete description: incorporated typical original participant remarks to characterize the phenomenon under study thoroughly. ⑥ We created the fundamental framework to entail grouping-related themes and descriptions, performed iterative comparisons, drew similar viewpoints, and developed topics. ⑦In validating the underlying structure, participants were given the theme framework of the results to validate. In the case of any deviations, the researcher would have to restart the study.

### Qualitative rigor

To establish credibility, both researchers analyzed the data. One graduate student who was not involved in the study and was familiar with qualitative research methods was invited during the data analysis phase to independently read the transcribed data and refine the themes to ensure the objectivity and authenticity of the study. Discussions were used to select the controversial aspect of topic refinement among three scholars. To ensure dependability, all respondents were asked to confirm the topic results, and they all agreed with the results. While working on this manuscript, the Consolidated Criteria for Reporting Qualitative Studies guidelines (COREQ) were followed [22].

### Ethics approval and consent to participate

The Ethics Committee of the Eye Hospital of Wenzhou Medical University approved the study (Ethical approval number: 2022-223-K-177-01). All respondents received an informed consent form via email, which they were required to read in detail and sign. Each participant was assigned a number to maintain anonymity, and all information collected was kept strictly confidential.

## Results

A total of 15 NGNs from four hospitals, aged between 22 and 24 years old, 3 males and 12 females, all with a bachelor’s degree, were interviewed for this study, as presented in Table 2. Four main categories and 13 subcategories were derived from the study data and presented in Table 3. To represent the views of NGNs, the following explanations and direct quotes (Chinese translation) describe the meaning of each theme.

**Table 3:** Main categories and subcategories of studies

Main categories	Subcategories
Perceptions and attitudes	Discovering the sparkle and recognizing oneself
	Integrating into new environments and enhancing adaptability
	Patient Safety
Drivers	Self-motivation
	Friendly and disciplined leader
	The Fascination of Positive Feedback
	An “involution” organizational climate
Dilemmas and needs	Negative feedback ---emotion regulation, communication
	Feedback environment --- real and open
	Feedback avoidance ---weakening the “face” culture
	Feedback channels --- diversity
Transformation and growth	Motivation shift Enhanced responsibility

### Theme 1: Perceptions and attitudes

NGNs had appropriate perceptions and positive attitudes toward feedback seeking. They perceived feedback as assisting them in better recognizing themselves, discovering personal strengths, facilitating better integration into the environment, enhancing adaptability, and assisting in maintaining patient safety.

#### Discovery of the sparkle and recognition of oneself

Feedback seeking can help NGNs discover their strengths and abilities to recognize better and improve themselves.

*“Some people do not know their strengths. In communication with others, others provide feedback and objective evaluation to help them discover that they did well. In this area, we may be able to receive unexpected gains. For example, to discover some of their abilities. ”(*N2).

*“One seeking feedback may do so because one wants to make progress or to have an objective understanding of one’s abilities or other aspects to seek external feedback.”* (N12).

#### Integrating into new environments and enhancing adaptability

Feedback-seeking behaviors help NGNs integrate into their new work environment and facilitate role adaptation.

*“Since some clinical supervisors have different work habits, one must adapt to various teachers. They can be exclusive. One could seek to integrate them through feedback. ”* (N9).

*“Clinical supervisors may have encountered the same problems as I did, and seeking help and feedback from them will enable me to better integrate into my new environment and adapt to my current life and work.”* (N3).

#### Patient safety

NGNs’ courage to seek feedback protects patient safety and reduces complications.

*“I feel that after I have tightened my grip on the detailed content of my work, I can effectively avoid some safety problems in caring for patients.”* (N8).

*“If you do not seek feedback, some problems may not be found or solved. The backlog remains, and minor problems will become big problems. At some point, you may not be able to solve them. However, if problems are addressed early, it will reduce the risk of harm to the patient.”* (N4).

### Theme 2: Drivers

The facilitators and drivers of FSB among NGNs were NGNs ' self-motivation to improve, upbeat leadership style, positive feedback, and an organizational climate of “involution,” where “involution” in Chinese culture refers to a situation and state of intense competition and secret competition among internal members.

#### Self-motivation

NGNs are motivated to seek feedback by their high expectations of themselves and desire for positive growth.

*“For example, as a new graduate nurse working at a more specialized psychiatric hospital, I cannot accomplish anything without prior exposure to such an environment if I do not seek feedback actively.”* (N7).

*“Other people’s evaluation of you may be different from what you think of yourself. For example, you may think you have done well in something, but your lead teacher’s opinion of you actually depicts that you are still far from adequate.”* (N11).

*“I think I am a person who seeks perfection, and I may ask myself to do my best not to make mistakes in my work. I take the initiative to seek help and feedback from colleagues or teachers when I encounter difficulties or do not handle things perfectly. ”*(N9).

#### A friendly and disciplined leader

Leaders who are rigorous and conscientious in their work and who are not distant are more likely to motivate NGNs to seek feedback.

*“A leader who gets along with me as a friend, without a sense of distance, will make me want to seek feedback more.”* (N9).

*“Severe leadership will be more demanding. The entire section of colleagues will be much more serious when doing things and try to use the leadership requirements to meet the standards. Under that kind of supervision, I try to reflect on myself as much as possible and increase the number of ward rounds.”* (N8).

*“If too severe or too fussy, it does not feel so good …… Not so friendly along, I will be slightly negative, not very active to seek ……”* (N7).

#### The fascination with positive feedback

Positive feedback from clinical instructors or leaders inspires the following feedback from NGNs.

*“If you get the teacher’s approval, you will be better at work. You will be motivated because you will have a sense of value when you receive approval from your colleagues, and you will be integrated into the process.”* (N5).

*“I am happy to receive positive feedback, and my clinical supervisors do not give me particularly negative feedback but generally encourage me a little.”* (N6).

*“The hospital is a high-pressure environment, so I believe healthcare workers still demand more positive feedback. ”*(N2).

#### An “involution” organizational climate

The organizational scope of “involution” refers to the current competitive work environment, which pushes NGNs to seek feedback to benefit from their careers.

*“Now the nursing industry is competitive. Of course, we still have to work harder privately, and I do not want to be the best, not too far from everyone, right? I always ask the teachers how things are going? Is this the right way to write the paperwork?”* (N7).

*“With the support of my peers, I will be more active and motivated because there is no contrast for one person, but three people will have a very sharp contrast.”* (N10).

*“I will want to adapt to the job when I enter this environment…… Although it will be a kind of pressure, it is also a kind of power to motivate me to work hard. After working in the daytime, I will go home and think about studying in private alone, which is also a kind of motivation.”* (N5).

*“In the workplace, hospitals have regular assessments. The content of the test is to penetrate the details of daily work, such as verifying patient identity and medication specifications. This will prompt me to understand and seek feedback on my shortcomings in my regular work……”* (N1).

### Theme 3: Dilemmas and needs

Throughout the feedback-seeking process, NGNs struggle with negative feedback, misleading and closed feedback environments, single feedback pathways, and self-feedback avoidance while also demonstrating their demands, according to the interviews.

#### Negative feedback: emotion regulation and communication

Receiving negative feedback is one of the dilemmas that NGNs need to face during the feedback-seeking process, and they need to regulate their emotions and communicate effectively to change such adverse situations.

*“Receiving negative feedback will be disappointing for me. It will question why I still do things wrongly after several feedbacks from teachers. If I still need to be reminded to do things rightly, I will lose confidence and doubt my ability to do things rightly. At this time, I hope to communicate more with the teacher. ”*(N12).

*“We are now in a state of identity shift, which affects our section assignments. Therefore, negative feedback can affect whether we are ultimately chosen by a good section.”* (N4).

*“Using negative feedback to gain growth is challenging but may work. However, one may have to endure the challenging times. The discomfort associated with this approach during such times can only be slowly eased through self-regulation. ”*(N2).

#### Feedback environment --- honest and open

False and closed feedback environments create difficulties in the feedback-seeking process for NGNs; therefore, there is a need to change adverse feedback environments in pursuit of truthfulness and openness.

*“In an inauthentic evaluation environment, sometimes we may misunderstand ourselves, believing that we are well, but in fact, there would still be room for improvement, which also affects our motivation to seek feedback because everyone may seem to be well.”* (N8).

*“However, our evaluation at the clinical section does not seem publicly available at the moment, so we do not know what other areas we can improve in the next section.”* (N1).

*“I would like to conduct the feedback less formally and deliberately and in a comfortable chatting format in a natural setting to show the true evaluation better.”*(N15).

#### Feedback avoidance ---weakening the “face” culture

The traditional Chinese culture of “face” creates feedback avoidance, further hindering NGNs from seeking feedback.

*“In the future, when you go to work independently and have problems, all the teachers will look down upon you, and I will have a psychological burden and may be more embarrassed to seek feedback. ”*(N4).

*“I was a student before I joined the hospital, but now I am a member of society, I am afraid to face interpersonal relationships in society because I have not been assigned to a department. Now I have no sense of belonging, and sometimes I do not want to seek feedback.”* (N6).

*“Some clinical instructors can make me feel oppressed rather than welcome, and I do not want to communicate with them as much. ”*(N11).

#### Feedback channels --- diversity

The current single feedback pathway has become challenging to facilitate seeking feedback from NGNs, and there is a need to pursue diverse feedback pathways.

*“The clinical instructors have their commitments and may not be able to spare more time for face-to-face communication with us, as they take care of their own families. ”*(N1).

*“The written form is a more euphemistic way. Sometimes, face-to-face interactions can be awkward, and you may not say what you think. However, if the interaction is written, it is easier to express suggestions, which is more acceptable by everyone.”* (N8).

*“If possible, I hope to build a platform for people to communicate with each other, but this platform should preferably be anonymous. ”*(N3).

*“The nursing manager has set up a small group of nurses in our department. In this group, clinical instructors will often give feedback on small challenges encountered during clinical practice.”* (N4).

### Theme 4: Transformation and growth

NGNs undergo a shift in feedback motivation as they transition from nursing students to credentialed nurses. They also show a stronger sense of responsibility in their work, which shows growth.

#### Motivation shift

NGNs also bring increased responsibility as their roles change, especially regarding patient care and personal future development.

*“In the past, I paid more attention to the final grade given by the teacher, which may affect whether you can stay here to work or not. Now, I would be more flexible in giving feedback on all aspects but pay more attention to what your leaders and colleagues think of you, especially in terms of interpersonal feedback.”* (N10).

*“As a student, my teachers were usually very proactive in pointing out irregularities in my nursing practice. Now that I am working, most of the operations are done on my own, and I am less likely to be watched, so I need to seek feedback on my own actively.”* (N3).

*“In the past, I would like to get feedback on the degree of knowledge mastery and academic performance, but after working, I would like t more feedback from your teachers on your ability to work. ”* (N14).

#### Enhanced responsibility

The changing role of the NGNs also brings with it an increased sense of responsibility, especially in terms of patient care and personal future development.

*“As an intern, I rarely sought feedback and would be more spontaneous. Now, I want to seek feedback more to improve my work and make my patients as happy as possible.”* (N1).

*“I need to be more accountable to patients, and I also feel that this approach will help me integrate into the environment more quickly and develop myself. ”*(N11).

*“In the past, I had a teacher who protected me from problems, and my main task was to familiarize myself with the workflow. I think I can store up my knowledge to use later in my work and in patient communication. ”*(N5).

*“The mutual output of the work, both my own and each other’s, is more informative, valuable, and educational than before, and a sign of responsibility to each.“*(N13).

## Discussion

### The significance of feedback-seeking deserves attention

The results of this study identified NGNs’ perceptions and attitudes toward FSBs, dilemmas, and needs, as well as transformation and growth in the feedback process. These open-ended responses complemented the blank components of the quantitative study. Through interviews with NGNs, we understood the significance of feedback seeking. It can help NGNs explore their strengths, better adapt to new environments, and contribute to patient safety.

Seeking feedback enables learners to receive and use information about their performance or behavior, contributing to more profound reflection on self and personal behavior [23]. Therefore, NGNs can seek feedback to reflect on themselves and identify their strengths and weaknesses. Feedback-seeking leads to better adaptation to new environments. This finding was consistent with a study [24], indicating that incorporating humanistic care into nursing management and creating a friendly environment to motivate FSBs can increase adaptation and reduce role maladaptation among junior nurses. Feedback-seeking promotes patient safety. This is partially consistent with findings from a study [25]. Zhang et al. concluded that feedback-seeking could influence safety performance through the feedback environment. The effect of feedback-seeking on patient safety can be facilitated by improving the feedback environment for NGNs. Therefore, hospitals and nursing administrators are called upon to pay adequate attention to NGNs’ FSB and maintain their positive attitudes and perceptions of feedback-seeking.

### Emphasis on feedback-seeking drivers

This study showed that feedback-seeking drivers include self-motivation, leadership style, positive feedback, and an “involutional” organizational climate.

Gan et al. argue that individual motivation is the main factor influencing FSB. The more motivated individuals are, the more actively they seek feedback from the environment [26]. Individuals’ motivations for seeking feedback broadly fit the self-motivation framework, and individuals seeking workplace-based assessment (WBA) feedback were associated with self-improvement and self-validation [27]. Interviews revealed that some NGNs seek feedback out of motivation to improve things or achieve self-improvement. As one of the primary sources of feedback, superiors play an essential role in employees’ FSB. Empirical studies have shown that the influence of leadership style on FSB is particularly prominent, and an upbeat leadership style can promote employees’ FSB [28]. For example, learning and ethical leaders can positively influence FSB [15, 29]. As mentioned in this study, approachable and disciplined leaders are similar in this regard and practice an upbeat leadership style. Leaders or managers can use their leadership style to improve outcomes among NGNs [30]. In the early stages of their careers, NGNs often do not have a well-defined plan for their future. They require a noble leadership style that promotes learning, stimulates intrinsic motivation and high-level pursuits, actively seeks feedback, and enables proactive behaviors critical to their professional growth and organizational development.

### Utilizing positive feedback and adapting to adversity

Positive feedback can promote feedback-seeking. Some NGNs felt that positive feedback would make them feel embedded within the organization and find a sense of value that would further promote feedback seeking.

Subordinates’ cognitive and affective-based trust in supervisors can influence their FSB [31]. Therefore, managers must provide NGNs with enough trust to encourage their active FSB through honest and objective positive feedback [32]. In doing so, NGNs can perceive their leaders’ trust and support for them and thus increase their organizational identity. Interviews revealed that NGNs are in a highly competitive organizational environment, commonly called “an involution.” In such environments, NGNs compete secretly and actively seek feedback to achieve better performance and assessment results in response to the hospital’s assessment and the efforts of those around them. The stronger the adversity-resistant mentality of NGNs, the more they seek measures to solve problems by improving and enhancing their professional abilities in the face of clinical stress and problems, thus consolidating the feedback-seeking process [33]. Hospitals or nursing managers should strengthen the training of NGNs to resist adversity and enhance their ability to adapt to adversity through real-life case studies, situational simulation exercises, and lectures. With that, they can adapt to the current “involution” environment and make better use of this environment, which is both pressure and motivation to seek feedback and promote professional growth.

### Careful use of negative feedback and openness to diverse feedback pathways

This study discovered that NGNs might receive negative feedback and fail to pursue an objective and authentic feedback environment, feedback avoidance, and a single feedback channel in the feedback-seeking process.

Some of the NGNs indicated in the interviews that negative feedback brings discomfort, such as stress and lack of confidence, which can seriously affect the motivation and initiative of NGNs in their work. However, it was found that more social support and increased implicit competence buffered the adverse effects of negative feedback on self-efficacy [34]. Therefore, emotional regulation, enhanced communication, and social support are needed to alleviate the stress caused by negative feedback among NGNs. Although negative feedback can sometimes be helpful [35], providers should provide problem-specific feedback, pay attention to the manner of negative feedback, and avoid overly harsh negative feedback.

Chinese traditional “face” culture affects the search for feedback. In taking care of the “face” of others, they will often be more cautious about negative feedback and sometimes provide untrue evaluations, impeding the essence of feedback and causing nurses to give up the initiative to seek feedback. This is consistent with the low quality of feedback and the leniency bias, that is, the reluctance of feedback providers to provide negative feedback, mentioned in the relevant literature review [36]. The feedback environment established by mentors can influence the FSBs of NGNs. When mentors create a favorable environment for feedback, NGNs’ professional adjustment can be enhanced by seeking feedback [37]. However, NGNs may give up these FSBs for fear of ridicule or undermining. Managers need to help NGNs overcome psychological barriers to a feedback-seeking and hierarchical culture that hinders bidirectional feedback [38]. Some scholars [39] argue that by establishing a culture of safe and fair feedback to facilitate learner feedback seeking, nursing managers need to establish an authentic, open, and fair feedback environment for NGNs. This can be achieved by lowering the threshold for seeking and providing feedback through specific activities, increasing the frequency of feedback conversations [40], creating an excellent organizational communication climate, establishing peer feedback, and encouraging exchanging work experiences and clinical expertise among colleagues. This provides objective and authentic feedback in a relaxed environment. Additionally, the entry qualifications of clinical instructors should be standardized to emphasize professional competence and focus on developing the willingness and skills to give feedback and meet the feedback needs of NGNs.

The feedback channels discussed in this paper are relatively homogeneous. However, in-person or written communication is known to have gradually failed to meet the needs of NGNs for feedback. They prefer new group chats or platforms to complete their feedback. Research has indicated the use of applications to quantitatively assess and reflect trainers’ propensity and desire for feedback in technical operations [41], suggesting feedback workshops, specific training, and the creation of supportive environments for feedback interactions to motivate individuals to actively seek feedback, and improve performance in the clinical workplace [42, 43]. Consequently, hospitals can first develop applications to assess the feedback needs of NGNs and then create a supportive environment for feedback interactions through feedback-seeking platforms and workshops to motivate NGNs’ FSBs.

### Enhancing feedback education and facilitating role transition

The latest interview findings revealed that NGNs experienced a change in feedback motivation between their time as nursing students and nurses.They demonstrated greater accountability by seeking feedback than the internship. NGNs will pay more attention to feedback on interpersonal relationships, such as leadership, co-workers, and work competencies. They will demonstrate a high level of responsibility both for self-development and patient care.

Therefore, to ensure the quality and safety of medical care, hospitals or nursing managers must pay attention to the feedback needs of NGNsand provide timely help and organizational support [44]. Concurrently, NGNs need to be appreciated or recognized for their positive and responsible work attitude and given enough psychological security and trust to facilitate their FSB as appropriate. Additionally, a thought-provoking issue was the lack of willingness to provide feedback on clinical competence and the lack of responsibility due to excessive psychological dependence on clinical supervisors when some NGNs were nursing students. NGNs are the future of nursing, and the education they receive as they transition into the workforce as new registered nurses is critical to stabilizing the nursing workforce, which will help maintain patient safety [45, 46]. Therefore, it is recommended that feedback learning education for nursing students be strengthened, feedback training programs be conducted, and the art and skill of seeking feedback be explored to enhance feedback self-confidence and promote proactive values. During the internship, independent patient management is learned to reduce psychological dependence on clinical supervisors.

### Implication for research and practice

This study sought to explore the feedback-seeking dilemmas and needs of NGNs to help nursing administrators and educators identify the undesirable effects of negative feedback, how to motivate NGNs to give feedback and to provide strategies and suggestions for future enhancement of FSBs of NGNs. The results of this study demonstrate the vital role of feedback in maintaining patient safety and stabilizing the nursing workforce in the context of global care. In addition, administrators and educators in other disciplines can trigger thinking about feedback to help better facilitate teaching and management.

### Limitations

The sample selected was only NGNs in Zhejiang Province, China, and was predominantly in a hospital setting; in the future, a larger, representative sample of NGNs in different settings, such as communities and clinics, will be needed to ask for feedback on their experiences of seeking; Foremost, the participants only had undergraduate education. In the future, we will consider the perspectives of NGNs with various levels of education, such as specialists and postgraduates. Eventually, the change in feedback motivation of NGNs lacks dynamic change observation, and further in-depth and comprehensive studies can be conducted.

## Conclusions

This study examined the FSBs of NGNs using interviews. First, NGNs recognized the critical role of feedback-seeking. Additionally, they found self-motivation, leadership style, positive feedback, and an organizational climate of “involution” to facilitate their initiative to seek feedback. Second, NGNs encountered struggles seeking feedback, which warrants in-depth exploration and requires appropriate measures to fulfill their feedback needs. Finally, we identified a positive shift in feedback motivation and growth in accountability among NGNs. This study provides suggestions and reflections for nursing administrators and educators to develop appropriate guidance and support to improve FSB among NGNs. Further future research is needed to explore the importance of feedback in the context of global care and to validate the applicability of the feedback strategies presented in this manuscript.

## Data Availability

The data presented in this article cannot be shared as the participants did not consent to sharing their responses. Requests regarding the datasets should be directed to the first author.

## Abbreviations

NGNs: New graduate nurses
FSB: Feedback-seeking behavior
